# Serum microRNA in patients undergoing atrial fibrillation ablation

**DOI:** 10.1038/s41598-020-61322-6

**Published:** 2020-03-10

**Authors:** Marek Kiliszek, Karolina Maciak, Agata Maciejak, Krystian Krzyżanowski, Robert Wierzbowski, Monika Gora, Beata Burzynska, Agnieszka Segiet, Andrzej Skrobowski

**Affiliations:** 1 Department of Cardiology and Internal Diseases, Military Institute of Medicine, Warsaw, Poland; 2 Institute of Biochemistry and Biophysics, PAS, Warsaw, Poland; 3 Department of Clinical Chemistry and Laboratory Diagnostics, Medical University of Warsaw, Warsaw, Poland; 4 Chair and Department of Experimental and Clinical Physiology, Medical University of Warsaw, Warsaw, Poland

**Keywords:** Interventional cardiology, Genetic markers

## Abstract

MicroRNAs mediate posttranscriptional gene regulation. The aim of the study was to find a microRNA predictor of successful atrial fibrillation (AF) ablation. A total of 109 patients undergoing first-time AF ablation were included. Nineteen patients were selected to undergo serum microRNA sequencing (study group). The sequencing data were used to select several microRNAs that correlated with 12-month recurrences after AF ablation. Those microRNAs were validated by digital droplet PCR in samples from remaining 90 patients. All patients underwent pulmonary vein isolation (RF ablation, contact force catheter, electroanatomical system). The endpoint of the study was the 12-month AF recurrence rate; the overall recurrence rate was 42.5%. In total, levels of 34 miRNAs were significantly different in sera from patients with AF recurrence compared to patients without AF recurrence. Six microRNAs (miR-183-5p, miR-182-5p, miR-32-5p, miR-107, miR-574-3p, and miR-144-3p) were validated in the whole group. Data from the validation group did not confirm the observations from the study group, as no significant differences were found between miRNAs serum levels in patients with and without recurrences 12 months after AF ablation.

## Introduction

Atrial fibrillation (AF) is one of the major causes of stroke, heart failure, sudden death, and cardiovascular morbidity in the world and is recognized as an independent factor that increases all-cause mortality. Effective treatment of AF and its symptoms can be attained by AF ablation, also known as pulmonary vein isolation (PVI). The major problem with this invasive treatment is that it is associated with a relatively high rate of AF recurrence. Several factors that influence the risk of recurrence have been found through laboratory tests and range from genetic factors to atrial fibrosis^1–3^.

A class of gene regulators that may be involved in AF recurrence is microRNAs (miRNAs). These are small (20–25 nucleotides long), single-stranded, nonprotein-coding RNAs that function as negative regulators of specific mRNA targets^4^. They promote target mRNA degradation and/or inhibit translation by sequence-specific base pairing. Accordingly, miRNAs are viewed as regulators of the expression of genes directly or indirectly involved in important physiological and pathophysiological processes. One advantage of miRNAs is that they can be detected in the circulation, making them attractive as potential biomarkers for the diagnosis and prognosis of diverse diseases^5^.

Our hypothesis in the present study was that serum miRNAs may reflect factors that significantly influence the risk of AF recurrence after catheter ablation. The aim of the study was to identify a miRNA predictor of successful pulmonary vein isolation in patients with AF.

## Results

### Clinical characteristics of patients

A total of 109 patients was included. The study group consisted of 19 patients, and the validation group consisted of 90 patients. The characteristics of the study and validation groups are shown in Table 1. The one-year recurrence rate was 42.2% (46 patients out of 109), 39% (30/77 patients) in the paroxysmal AF subgroup, and 50% (16/32 patients) in the non-paroxysmal AF subgroup.

**Table 1:** The characteristics of the study and validation groups.

	Study group (n = 19)	Validation group (n = 90)	p value
Age	63 (58-67)	63 (56-68)	0.97
Women/men	5/14	37/53	0.34
Non-paroxysmal AF	9 (47.4%)	38 (42.2%)	0.88
Hypertension	13 (68.4%)	68 (75.6%)	0.72
Diabetes	6 (31.6%)	17 (18.9%)	0.36
Coronary artery disease	2 (10.5%)	9 (10.0%)	0.73
Left ventricular ejection fraction	60 (54-60)	60 (56-65)	0.12
Duration of AF ablation (minutes)	127 (16)	127 (24)	0.94
Duration of RF applications (seconds)	2395 (533)	2146 (587)	0.09
12 months recurrence	8 (42.1%)	38 (42.2%)	0.81

Data are shown as median (Q1–Q3) or mean (standard deviation) or number (%). Student’s t-test was used for normally distributed variables and the Mann-Whitney test otherwise. Comparisons of categorical variables were made using chi-square test or Fisher’s exact test.

### Identification of differentially expressed miRNAs by next generation sequencing

A pilot-scale study was performed to screen circulating miRNA profiles in patients with recurrent AF (n = 8) and in patients with no AF recurrence (n = 11). Levels of serum miRNAs were determined by high-throughput small RNA sequencing. An average of 25.8 million reads was obtained per sample. The distribution of mappable reads was within the normal range and similar for all samples. The number of identified known miRNAs was 496 with a number of counts ≥1 tags per million (TPM) and 270 with ≥10 TPM in all samples.

In total, 34 miRNAs showed significantly altered serum levels in patients with AF recurrence compared to patients without AF recurrence (Table 2). Among these, 29 miRNAs were downregulated and 5 miRNAs were upregulated. Gene ontology (GO) enrichment analysis was performed to identify GO terms that were significantly associated with the target genes of the dysregulated miRNAs. Several biological processes related to the pathophysiology of the cardiovascular system were enriched, the most important being regulation of heart morphogenesis (GO:2000826, p = 0,00241), positive regulation of cardiac muscle cell differentiation (GO:2000727, p = 0,00522), cardiac conduction (GO:0061337; p = 0,01163), ion membrane transport (GO:0034220, p = 0,01257), and regulation of the heart rate by chemical signals (GO:0003062, p = 0,01968).

**Table 2:** Differentially expressed miRNAs obtained by small RNA sequencing in patients with AF recurrence compared to those without AF recurrence.

miRNA^a^	Sequence	Log_2_FC^b^	*p*-value
**hsa-miR-184**	**TGGACGGAGAACTGATAAGGGT**	**−2.574**	**0.00001**
**hsa-miR-183-5p**	**TATGGCACTGGTAGAATTCACT**	**−0.908**	**0.00089**
**hsa-miR-182-5p**	**TTTGGCAATGGTAGAACTCACACT**	**−0.836**	**0.00271**
hsa-miR-484	TCAGGCTCAGTCCCCTCCCGAT	−0.700	0.00283
**hsa-miR-32-5p**	**TATTGCACATTACTAAGTTGCA**	**−0.619**	**0.00344**
hsa-miR-92b-3p	TATTGCACTCGTCCCGGCCTCC	−0.674	0.00385
hsa-miR-1224-5p	GTGAGGACTCGGGAGGTGG	−0.831	0.00412
hsa-miR-92a-3p	TATTGCACTTGTCCCGGCCTGT	−0.556	0.00488
hsa-miR-451a	AAACCGTTACCATTACTGAGTT	−0.580	0.00718
**hsa-miR-107**	**AGCAGCATTGTACAGGGCTATCA**	**−0.470**	**0.00807**
hsa-miR-375	TTTGTTCGTTCGGCTCGCGTGA	−0.853	0.00880
**hsa-miR-574-3p**	**CACGCTCATGCACACACCCACA**	**0.563**	**0.00895**
hsa-miR-22-3p	AAGCTGCCAGTTGAAGAACTGT	−0.452	0.01205
**hsa-miR-203a**	**GTGAAATGTTTAGGACCACTAG**	**−1.351**	**0.01459**
hsa-miR-548d-5p	AAAAGTAATTGTGGTTTTTGCC	−0.725	0.01599
hsa-miR-3158-3p	AAGGGCTTCCTCTCTGCAGGAC	−0.779	0.01651
hsa-miR-551a	GCGACCCACTCTTGGTTTCCA	0.815	0.01867
hsa-miR-16-2-3p	CCAATATTACTGTGCTGCTTTA	−0.581	0.01908
hsa-miR-339-5p	TCCCTGTCCTCCAGGAGCTCACG	0.393	0.02031
**hsa-miR-141-3p**	**TAACACTGTCTGGTAAAGATGG**	**−0.823**	**0.02254**
hsa-miR-423-5p	TGAGGGGCAGAGAGCGAGACTTT	−0.376	0.02407
**hsa-miR-144-3p**	**TACAGTATAGATGATGTACT**	**−0.672**	**0.02416**
hsa-miR-486-3p	CGGGGCAGCTCAGTACAGGAT	−0.543	0.02451
hsa-miR-18a-3p	ACTGCCCTAAGTGCTCCTTCTGG	−0.609	0.02534
hsa-miR-1180-3p	TTTCCGGCTCGCGTGGGTGTGT	−0.492	0.02569
hsa-miR-1299	TTCTGGAATTCTGTGTGAGGGA	−2.055	0.02954
hsa-miR-196b-5p	TAGGTAGTTTCCTGTTGTTGGG	−0.516	0.03003
hsa-miR-215-5p	ATGACCTATGAATTGACAGAC	−0.537	0.03117
hsa-miR-1246	AATGGATTTTTGGAGCAGG	−0.518	0.04176
hsa-miR-363-3p	AATTGCACGGTATCCATCTGTA	−0.581	0.04348
hsa-miR-21-5p	TAGCTTATCAGACTGATGTTGA	−0.369	0.04765
hsa-miR-145-5p	GTCCAGTTTTCCCAGGAATCCCT	0.570	0.04905
hsa-miR-326	CCTCTGGGCCCTTCCTCCAG	0.368	0.04910
hsa-miR-1294	TGTGAGGTTGGCATTGTTGTCT	−0.512	0.04977

^a^miRNAs selected for subsequent validation are indicated in bold. ^b^Logarithm of Fold Change (FC).

### Validation of selected dysregulated miRNAs using ddPCR

We selected nine candidate miRNAs that differentiated the patients with AF recurrence from patients without AF recurrence, based on the statistical significance, fold change difference, individual TPM values, and a literature search. The selected miRNAs were miR-184, miR-183-5p, miR-182-5p, miR-32-5p, miR-107, miR-574-3p, miR-203a, miR-141-3p, and miR-144-3p. The validation was carried out by ddPCR on serum samples of the independent group consisting of patients with recurrent AF (n = 38) and patients with no AF recurrence (n = 52).

**Figure 1:**
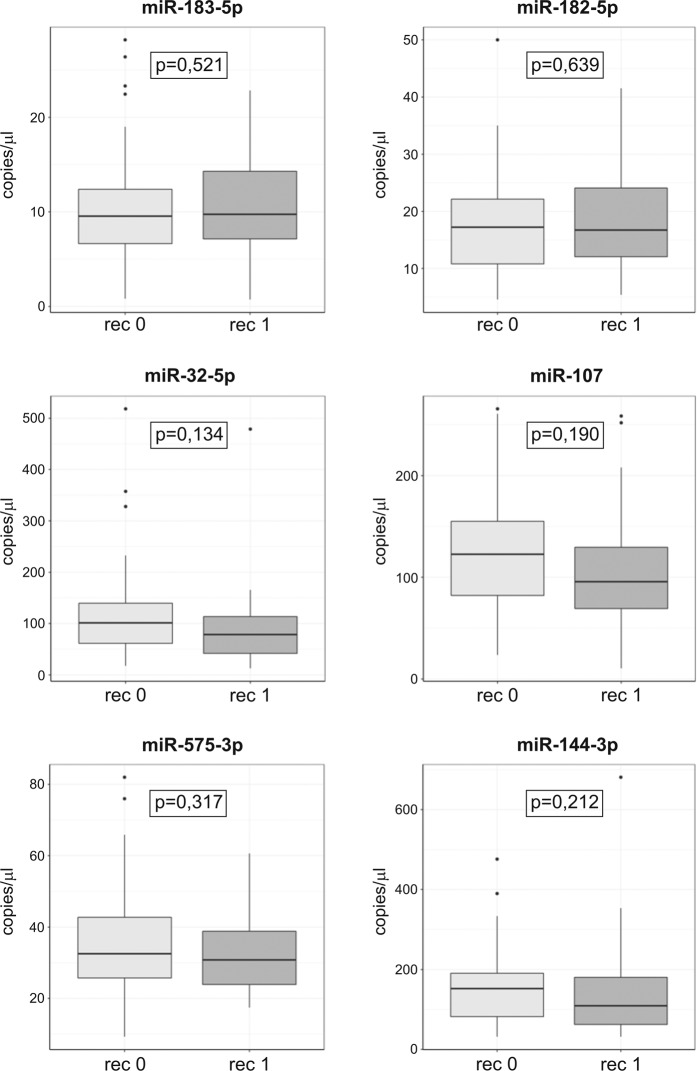
Serum levels of selected miRNAs validated by ddPCR.Box plots show the levels of miRNAs (in copies per microliter of serum). The bottom and top of each box presents the 1st and 3rd quartiles of the data, respectively. The median is presented as a solid line across the box. Dots represent outliers. The distribution of variables between groups was compared using Student’s t-test for normally distributed variables and the Mann-Whitney test otherwise. rec 0 -patients without AF recurrence (n = 52); rec 1 - patients with AF recurrence (n = 38).

The miRNAs miR-184, miR-203a, and miR-141-3p were excluded from further analysis due to their very low target copy numbers and a lack of positive droplet cluster formation. Consistent with the sequencing data, miR-32-5p, miR-107, and miR-144-3p were downregulated in sera from patients with AF recurrence compared to patients without AF recurrence, but the differences in expression did not reach statistical significance. The results of ddPCR validation did not confirm a selective upregulation of miR-574-3p or downregulation of miR-183-5p and miR-182-5p (Fig. 1). No statistical significance was observed for miRNA serum levels between patients with and without recurrences in the paroxysmal and non-paroxysmal subgroups of AF patients (data not shown). Fourteen patients during follow-up underwent one or more re-do procedures, and 6 patients had AF despite isolated pulmonary veins. None of the miRNAs tested was significantly different in sera from patients with AF recurrences despite isolated pulmonary veins (data not shown).

## Discussion

Our aim in this study was to find a miRNA predictor of successful AF ablation (pulmonary vein isolation). In the study group, we found several miRNAs whose levels were different in sera from patients with and without recurrences 12 months after AF ablation. However, validation of the 90-patient group did not confirm the results.

Several miRNAs has been proposed as biomarkers of AF, but none currently is available for diagnostic purposes^6^. The data on a link between miRNA and results of AF ablation are scarce. Participants with AF had lower plasma levels of miR-21 compared to controls, and miR-21 expression increased 1 month after catheter ablation^7^. The circulating miR-21 level is also associated with AF ablation results in patients with persistent AF undergoing ablation (and this level correlates with left atrial low voltage areas)^8^. Other studies have shown higher levels of miRNAs 125a, 10b, 601, 30a-3p, and 199b in patients with 12-month AF recurrences after catheter ablation than in patients without AF recurrences^9^.

Our results suggest that serum miRNAs lack any strong signals with a potential to predict AF ablation results, at least in the population consisting primarily of patients with paroxysmal AF. We made every effort to make the results reliable. The patient population was, whenever possible, consecutive, and AF ablation was conducted according to current guidelines^10^. The patient population in our groups was comparable to that reported in the German AF ablation registry and in the EORP AF ablation pilot registry^11,12^. Our results were comparable to those in the German AF ablation registry (the recurrence rate in our study was 42.1% during 1 year of observation vs. 45.9% in the German AF ablation registry).

Due to the small size and low abundance of miRNAs in body fluids, miRNA expression profiling is technically challenging. For this reason, next generation sequencing (NGS) has recently become a preferred method for miRNA profiling^13^. When compared with the standard quantitative real-time PCR, the NGS platforms are the most robust for comprehensive expression profiling of miRNAs and offer the greatest detection sensitivity, the largest dynamic range of detection, and the highest accuracy^14^.

Since ddPCR has demonstrated a high degree of linearity and quantitative correlation in measuring miRNAs^15^, we used this method to confirm the results from our small RNA sequencing experiments. The ddPCR is a method based on PCR. It enables a precise quantification of nucleic acids. The digital PCR has capability to obtain absolute quantification without external references that gives advantage over classical real-time PCR^16^.

The majority of patients with AF recurrences after AF ablation showed a reconduction of pulmonary veins during a redo procedure. At present, new electrophysiological tools are available (e.g., the Ablation Index) that decrease the number of recurrences (and probably, to some extent, the reconductions as well) to less than 10% in paroxysmal AF patients^17^. These results still require large multicenter validation. We were unable to find any significant miRNA biomarker in our entire population of patients with AF recurrences; therefore, future studies are needed to find predictors of AF recurrence despite the presence of isolated pulmonary veins. These biomarkers might be important in patient selection and in planning the AF ablation procedure. Our subgroup of patients with AF recurrences after AF ablation and isolated pulmonary veins in redo procedures did not show any significant differences in serum levels of the tested miRNAs, but the group was too small (n = 6) to draw any reliable conclusions.

## Strengths and Limitations

All the clinical procedures (patient qualifications, AF ablation, and follow-up) were performed according to the current guidelines. We performed miRNA sequencing in the study group to avoid a priori hypotheses, and validation was performed with ddPCR, which is one of the best methods available. Nevertheless, this was a single-center study with limited number of patients, most of whom showed paroxysmal AF. The miRNA sequencing was performed on a small study group (19 patients), and some miRNAs could not be verified due to very low serum expression.

In conclusion, our results suggest that the serum miRNA of patients with AF (majorly paroxysmal) does not contain any strong signals with the potential to predict AF ablation results.

## Materials and Methods

### Patients, blood sample collection and serum separation

This was a single-center, prospective study (index ablation operations conducted during the period of 2015–2016). Consecutive patients with atrial fibrillation (paroxysmal and non-paroxysmal) and undergoing their first PVI were enrolled. Exclusion criteria were a previous AF ablation and significant inflammatory disease. All patients had symptomatic drug-refractory AF and were qualified for PVI according to contemporary guidelines^10^. All subjects gave their informed consent for inclusion before participation in the study. The study was conducted in accordance with the Declaration of Helsinki, and the protocol was approved by the Bioethics Committee of the Military Institute of Medicine.

Blood was collected during venous puncture at the beginning of ablation, and centrifuged after clotting at 1500 × g for 10 min at room temperature. The resulting serum samples were transferred to cryogenic vials and stored at −80 °C until analysis.

The patient group was divided into study and validation groups. Serum samples from patients from the study group underwent miRNA sequencing. Candidate miRNAs differentiating the patients with AF recurrence from patients without AF recurrence were chosen based on the statistical significance, fold change difference, individual TPM values, and a literature search. The selected miRNAs were then validated by digital droplet PCR (ddPCR) of the validation group samples.

The primary study endpoint was freedom from recurrence of any atrial tachyarrhythmia (>30 s duration) at 12 months (after a blanking period of 3 months).

Ablation strategy. The left atrium was accessed through a double transseptal puncture, and a circumferential mapping catheter and irrigated contact force catheter were used for mapping and radiofrequency (RF) ablation. Fluoroscopy and an electroanatomical CARTO 3 system (Biosense Webster, Diamond Bar, CA, USA) were used to navigate the catheters. The temperature limit was 48 °C and the power limit was 30 W (25 W on posterior wall) while delivering the RF energy. The ipsilateral veins were isolated jointly. The endpoint of the procedure was isolation of all pulmonary veins. Cardioversion was performed, if needed, to verify the isolation during sinus rhythm. Applications other than PVI were performed only if the patient developed atrial tachycardia, typical or atypical atrial flutter, or frequent atrial ectopy.

Follow-up. Recurrence of AF was defined as any atrial tachycardia lasting more than 30 s with a three-months blanking period applied. All patients were recommended to discontinue antiarrhythmic drugs immediately after catheter ablation. The patients were scheduled for two follow-up visits after 6 and 12 months, and all asymptomatic patients underwent 7-day Holter monitoring (monitoring was not performed in patients with an already documented recurrence of AF).

### Library preparation, next generation sequencing, and data analysis

Small RNA sequencing experiments and data analysis were conducted by Qiagen Genomic Services (Hilden, Germany). RNA was extracted from 200 μL serum with the QIAseq miRneasy advanced RNA isolation protocol optimized for serum/plasma. Total RNA was eluted in ultra-low volume. The library was prepared using the QIAseq miRNA Library Kit (Qiagen, Hilden, Germany). A total of 5 µL total RNA was converted into miRNA NGS libraries. Adapters containing Unique Molecular Identifiers (UMIs) were ligated to the RNA, and the RNA was then converted to cDNA. The cDNA was amplified using PCR (22 cycles) and indices were added during the PCR. The samples were purified after the PCR. Library preparations were subjected to quality control using either a Bioanalyzer 2100 or TapeStation 4200 system (Agilent Technologies, Santa Clara, CA, USA). The quality of the inserts and the concentration measurements were used to pool the libraries in equimolar ratios. The library pools were quantified using the qPCR ExiSEQ LNA Quant kit (Exiqon, Vedbaek, Denmark) and sequenced on the NextSeq. 500 sequencing platform (Illumina Inc., San Diego, CA, USA). This yielded 75 nucleotide single-ended reads of good quality (Q score greater than 30). Raw data were demultiplexed and FASTQ files for each sample were generated using the bcl2fastq software (Illumina Inc.). FASTQ data were checked using the FastQC tool^18^. The adapters were trimmed off as part of the base calling. After filtering of the adapters, the samples had the expected main peak of around 18–23 nucleotides representing miRNAs. The sequencing reads were mapped to entries in miRBase release 20^19^.

The supervised analysis of expression levels was performed using the trimmed mean of M-values normalization method (TMM normalization) in the EdgeR statistical software package^20^. The miRNAs were considered to be significantly differentially expressed at a cut-off of p-value < 0.05, as estimated by an exact test on the negative binomial distribution. The Gene Ontology (GO) enrichment analysis was performed using miRSearch^21^ to identify target genes of the differentially expressed miRNAs. The standard Fisher’s test was used to investigate enrichment of GO terms between the groups, followed by the ‘Elim’ method incorporating the topology of the GO network to compensate for local dependencies between GO, which can mask significant GO terms^22^.

### MiRNA isolation and cDNA synthesis

Total RNA with preserved miRNAs was extracted from 200 μL of serum samples using the miRNeasy Serum/Plasma Advanced Kit (Qiagen) in accordance with the manufacturer’s protocol. To maximize the yield and reproducibility, 1.25 μg/mL of MS2 bacteriophage RNA carrier (Roche Diagnostics, Mannheim, Germany) was added at the beginning of the procedure. For quality control of the RNA isolation, three synthetic RNA spike-ins (UniSp2, UniSp4, and UniSp5) from the RNA Spike-in Kit for RT (Qiagen) were added to the samples prior to purification at concentrations recommended by the manufacturer. The cDNA was synthesized from purified miRNA according to the protocol supplied with the miRCURY LNA RT Kit (Qiagen), with the addition of two spike-ins (UniSp6 and cel-miR-39-3p) intended as a cDNA synthesis control.

### Droplet digital PCR

Selected miRNAs were subjected to a subsequent validation step by ddPCR using the QX200 Droplet Digital PCR system (Bio-Rad, Hercules, CA, USA). The cDNAs were diluted 10-fold for low-copy targets (hsa-miR-182-5p, hsa-miR-183-5p, and hsa-miR-574-3p) or 20-fold for the others, and 9 µL was used in each 20 µL of ddPCR reaction using miRCURY LNA miRNA PCR assays (Qiagen) and QX200 ddPCR EvaGreen Supermix (Bio-Rad). A no-template control (NTC) was included in every assay. The ddPCR reactions were partitioned and emulsified in 70 µL of QX200 Droplet generation oil for EvaGreen in the QX200 Droplet Generator (Bio-Rad). The resulting droplets were transferred to a 96-well plate and PCR was performed in the T100 Thermal Cycler (Bio-Rad) with the thermal cycling conditions recommended for EvaGreen assays. After amplification, the plates were loaded into the QX200 Droplet Reader and the ddPCR results were analyzed with QuantaSoft software v.1.7.4.0917 and QuantaSoft Analysis Pro software v.1.0.596 (Bio-Rad). The absolute number of target molecules in the ddPCR reaction was determined using Poisson statistical analysis and background-corrected based on the NTC. The concentration of target miRNA was further corrected for the input amount of serum, and the final results were provided as the number of copies per microliter of serum.

### Statistical analysis

Descriptive characteristics reported for continuous variables were medians with 1st and 3rd quartiles or mean (standard deviation), depending on normality of distribution of variable. Normality of variables distribution was tested using the Shapiro-Wilk test. Group comparisons were made using Student’s t-test for normally distributed variables and the Mann-Whitney test otherwise. Categorical variables were presented as frequencies, group comparisons were made using chi-square test or Fisher’s exact test. For all calculations, 2-tailed tests were used, and the level of significance was set at 0.05.

Statistical power of the study. Assuming a standard deviation of miRNA of about 50% of the mean value of that miRNA, we were able to detect a 1.3 fold change or greater with 80% power at the level of significance 0.05.

Statistical analysis was performed with Statistica v. 12 (Statsoft Inc., Tulsa, Oklahoma, United States) and R statistical software, version 3.4.0^23^.

## References

[1] Marrouche NF (2014). Association of atrial tissue fibrosis identified by delayed enhancement MRI and atrial fibrillation catheter ablation: the DECAAF study. JAMA..

[2] Jiang H, Wang W, Wang C, Xie X, Hou Y (2017). Association of pre-ablation level of potential blood markers with atrial fibrillation recurrence after catheter ablation: a meta-analysis. Europace..

[3] Rattanawong P, Chenbhanich J, Vutthikraivit W, Chongsathidkiet P (2018). A chromosome 4q25 variant is associated with atrial fibrillation recurrence after catheter ablation: A systematic review and meta-analysis. J. Atr Fibrillation.

[4] Bartel DP (2004). microRNAs: Genomics, biogenesis, mechanism, and function. Cell..

[5] Maciejak A (2018). Circulating miR-30a-5p as a prognostic biomarker of left ventricular dysfunction after acute myocardial infarction. Sci. Rep..

[6] Komal S (2019). MicroRNAs: Emerging biomarkers for atrial fibrillation. J. Cardiol..

[7] McManus DD (2015). Plasma microRNAs are associated with atrial fibrillation and change after catheter ablation (the miRhythm study). Heart Rhythm.

[8] Zhou Q (2018). Circulating microRNA-21 correlates with left atrial low-voltage areas and is associated with procedure outcome in patients undergoing atrial fibrillation ablation. Circ. Arrhythm. Electrophysiol..

[9] Vaze A (2017). Plasma microRNAs relate to atrial fibrillation recurrence after catheter ablation: longitudinal findings from the MiRhythm Study. J. Clin. Exp. Cardiol..

[10] Camm AJ (2012). 2012 focused update of the ESC Guidelines for the management of atrial fibrillation: an update of the 2010 ESC Guidelines for the management of atrial fibrillation–developed with the special contribution of the European Heart Rhythm Association. Europace.

[11] Sultan A (2017). Predictors of atrial fibrillation recurrence after catheter ablation: Data from the German Ablation Registry. Sci. Rep..

[12] Riahi S (2016). Regional differences in referral, procedures, and outcome after ablation for atrial fibrillation in Europe: a report from the Atrial Fibrillation Ablation Pilot Registry of the European Society of Cardiology. Europace.

[13] Coenen-Stass, A. M. L. *et al*. Evaluation of methodologies for microRNA biomarker detection by next generation sequencing. *RNA Biol*. **15**, 1133–1145. 10.1080/15476286.2018.1514236 PMC6161688 30223713

[14] Tam S, de Borja R, Tsao MS, McPherson JD (2014). Robust global microRNA expression profiling using next-generation sequencing technologies. Lab. Invest..

[15] Robinson S (2018). Droplet digital PCR as a novel detection method for quantifying microRNAs in acute myocardial infarction. Int. J. Cardiol..

[16] Taylor SC, Laperriere G, Germain H (2017). Droplet Digital PCR versus qPCR for geneexpression analysis with low abundant targets: from variable nonsense to publication quality data. Sci Rep.

[17] Phlips Thomas, Taghji Philippe, El Haddad Milad, Wolf Michael, Knecht Sébastien, Vandekerckhove Yves, Tavernier René, Duytschaever Mattias (2018). Improving procedural and one-year outcome after contact force-guided pulmonary vein isolation: the role of interlesion distance, ablation index, and contact force variability in the ‘CLOSE’-protocol. EP Europace.

[18] Babraham Bioinformatics FastQC. Available online: http://www.bioinformatics.babraham.ac.uk/projects/fastqc/ (accessed on 2 February 2018).

[19] Kozomara A, Griffiths-Jones S (2014). MiRBase: Annotating high confidence microRNAs using deep sequencing data. Nucleic Acids Res..

[20] Bioconductor. Available online: http://www.bioconductor.org/ (accessed on 15 November 2018).

[21] miRSearch. Available online: https://www.exiqon.com/mirsearch (accessed on 15 November 2018).

[22] Alexa A, Rahnenführer J, Lengauer T (2006). Improved scoring of functional groups from gene expression data by decorrelating GO graph structure. Bioinformatics.

[23] R Development Core Team. R: A Language and Environment for Statistical Computing, R Foundation for Statistical Computing, Vienna, Austria, http://www.R-project.org (2006).

